# Cyberchondria and complexity: a systems-level exploration of anxiety and informational instability in the digital age

**DOI:** 10.3389/fpsyg.2026.1794803

**Published:** 2026-03-19

**Authors:** Gabriella Martino, Maria Laura Giacobello, Orlando Silvestro, Alessandro Meduri, Giorgio Sparacino, Sebastiano Gangemi, Carmelo Mario Vicario

**Affiliations:** 1 Department of Clinical and Experimental Medicine, University of Messina, Messina, Italy; 2 Department of Ancient and Modern Civilisations, University of Messina, Messina, Italy; 3 Department of Health Sciences, University Magna Graecia of Catanzaro, Catanzaro, Italy; 4 Department of Biomedical and Dental Sciences and Morphofunctional Imaging, University of Messina, Messina, Italy; 5 Course Degree in Medicine and Surgery, Department of Adult and Childhood Human Pathology “Gaetano Barresi”, University of Messina, Messina, Italy; 6 Department of Cognitive Science, Psychology, Education and Cultural Studies, University of Messina, Messina, Italy

**Keywords:** AI-mediated symptom-checking, cognitive decoherence, complexity, cyberchondria, digital anxiety, health-related uncertainty, informational entropy, psychopathology

## Abstract

**Background:**

Cyberchondria, defined as excessive or compulsive online searching for health-related information, has emerged as a paradigmatic condition at the intersection of health anxiety, digital behavior, and cognitive vulnerability. Increasingly observed in clinical settings, it reflects a maladaptive strategy to manage uncertainty and bodily symptoms, exacerbated by algorithm-driven content, emotional salience, and informational overload in digital environments. Cyberchondria may have important implications for medical outcomes, including inappropriate healthcare utilization, reduced adherence to medical advice, disruption of the doctor-patient relationship, and impaired quality of life, especially in individuals managing chronic or recurrent health conditions.

**Aim:**

This article proposes an integrative framework for understanding cyberchondria that combines clinical psychology, cognitive science, and complexity theory. We argue that cyberchondria should not be viewed solely as an individual pathology, but as an emergent property of a dynamic system destabilized by recursive feedback loops, epistemic instability, and algorithmic bias.

**Methods:**

A narrative synthesis of empirical and theoretical literature was conducted, drawing from studies on health anxiety, online behavior, metacognition, and systemic models of mental disorders. Sources were selected from PubMed, Scopus, and interdisciplinary publications in psychiatry, cognitive science, and digital ethics.

**Results:**

Cyberchondria is consistently associated with intolerance of uncertainty, reassurance-seeking, and dysfunctional metacognitive beliefs. Its persistence is sustained by cognitive distortions and by the structure of digital health information, which prioritizes emotionally salient or sensational content over clinical reliability. The phenomenon can be theoretically modelled using concepts such as psychological entropy, nonlinear amplification, and cognitive decoherence under informational stress.

**Conclusion:**

Effective responses to cyberchondria must operate across multiple levels: psychological (CBT and metacognitive strategies), educational (digital health literacy), relational (doctor–patient alliance), and systemic (ethical design of digital platforms). Integrating complexity-based perspectives may offer new pathways for understanding and mitigating the destabilizing effects of cyberchondria in the digital age.

## 1 Introduction

The term cyberchondria, a portmanteau of “cyber” and “hypochondria,” refers to the escalating distress resulting from compulsive online health information seeking. The neologism was coined to describe a behavioral pattern in which the need for reassurance paradoxically perpetuates anxiety and worry, resulting in the misinterpretation of benign symptoms as indicators of serious illness. Although the expression was initially popularized in media outlets around 2001, it has since gained traction in the scientific literature as a clinically relevant construct, now widely recognized as a digital-age manifestation of health anxiety (White and Horvitz, 2009; Starcevic et al., 2019).

Beyond its psychic burden, cyberchondria represents a clinically relevant factor that may significantly affect disease management, adherence to medical treatments, and the utilization of healthcare services, thus positioning it within the broader framework of psychological determinants of medical conditions. Because the Web provides access to a broad range of medical information, online health-related searches may heighten anxiety among lay individuals; cyberchondria specifically denotes the unwarranted intensification of concerns about common symptoms following exposure to online content (White and Horvitz, 2009).

The exponential rise in online medical content, combined with algorithm-driven curation, anecdotal narratives, and unverified forums, has restructured how individuals interpret physical symptoms and health threats. Rather than providing clarity, this informational abundance often fuels intolerance of uncertainty, anxiety sensitivity, and obsessive-compulsive tendencies, all of which are robustly associated with cyberchondria (Schenkel et al., 2021; Yang et al., 2022).

Within this context, cyberchondria can be conceptualized as a relatively specific syndromic construct, distinct from other related constructs and composed of a set of interrelated symptoms (Starcevic et al., 2019). Among the most salient are problematic Internet use and health anxiety (Yang and Xu, 2025). Compulsive Internet use driven by health anxiety is significantly associated with the severity of cyberchondria, as documented by several studies (Khazaal et al., 2021; Arsenakis et al., 2021; Fergus, 2015). Additional factors commonly associated with this condition include, beyond intolerance of uncertainty, the need for exhaustive and perfectly coherent explanations of symptoms and other health issues, as well as selective attention to health-related information (Starcevic, 2017).

Moreover, it has been suggested that further investigation into the potential link between neuroticism, a personality trait consistently associated with problematic Internet use, and cyberchondria (Bajcar and Babiak, 2020).

It is worth underscoring that, regardless of whether health anxiety or online health-related searching occurs first, engaging in such searches within the context of cyberchondria exacerbates health anxiety beyond the levels experienced before initiating the search behavior. Longitudinal studies have shown a reciprocal relationship between online symptom searching and health anxiety, reinforcing a maladaptive feedback loop (te Fam Poel et al., 2016).

Considering this, cyberchondria can be understood as repeated online searching for health-related information associated with increasing levels of health anxiety. Moreover, individuals affected by cyberchondria tend to devote excessive time to such searches, thereby reducing their engagement in other social activities and giving rise to additional negative consequences (Starcevic, 2017).

In summary, cyberchondria appears to be characterized by repetitive symptom-checking, compulsive searching for medical information, and increased concern over vague or ambiguous bodily sensations. These behaviors tend to exacerbate rather than alleviate health-related anxiety, often leading to functional impairment (Starcevic et al., 2020).

Emerging data from the COVID-19 pandemic further highlight how perceived vulnerability, uncertainty, and increased digital engagement contribute to cyberchondria (Infanti et al., 2023). Particularly vulnerable groups include adolescents exposed to family dysfunction and low levels of dispositional optimism (Liu et al., 2022), as well as individuals who rely on unreliable sources of medical information (Bahadir and Dundar, 2024). Further research corroborates that elevated levels of cyberchondria among students are associated with increased problematic Internet use and health anxiety (Aşut and Gülbetekin, 2025; Jungmann and Dessauer, 2025; Gergin et al., 2025).

Despite growing academic attention, consensus on many of the defining features of cyberchondria has yet to be achieved, as the condition has not been sufficiently explored given its clinical relevance. Consequently, further research is warranted to identify key avenues for future investigation (Vismara et al., 2020), including its longitudinal trajectory and impact at both individual and societal levels.

The increase in cyberchondria is closely linked to the information overload introduced by the digital revolution, which poses challenges for managing online health information, particularly when such information is ambiguous or inconsistent. In this context, cyberchondria reveals distinct maladaptive patterns of interaction with the Internet, driven by unrealistic expectations about online resources and misconceptions about interpreting search results (Yang and Xu, 2025). This underscores the importance of examining problematic and dependency-related patterns of Internet use that may exacerbate cyberchondria and negatively impact overall well-being. The development of evidence-based guidelines to identify and address Internet-related addictive behaviors is essential to mitigate their harmful effects and to prevent unnecessary strain on healthcare systems. Given the widespread integration of the Internet into daily life and the potential adverse consequences of online health-related searches, cyberchondria may constitute a substantial public health burden. Furthermore, it represents a significant concern for bioethics, understood as the critical conscience of technological civilization (Pessina, 1999).

For these reasons, it may be imprudent to await a full conceptual consensus on cyberchondria or a more comprehensive understanding of the condition. Instead, efforts should focus on designing therapeutic approaches that can be evaluated in randomized controlled trials to support individuals who experience overwhelming anxiety during online health-related searches (Starcevic, 2017).

While clinical psychology has illuminated the cognitive underpinnings of cyberchondria, additional theoretical frameworks are needed to fully explain its persistence and amplification in the digital ecosystem. Conceptualizing cyberchondria as a complex interaction among psychological traits, informational environments, and algorithmic structures opens the door to interdisciplinary approaches. The application of information theory and complexity science, including notions such as informational entropy, nonlinear feedback, and emergence, may help explain why increased access to information can paradoxically generate greater uncertainty and cognitive disorganization (Olthof et al., 2020).

In this article, we offer an integrated overview of cyberchondria by examining its clinical and cognitive features, identifying the psychological mechanisms that sustain it, analyzing the influence of digital media and algorithmic systems, and exploring its potential characterization as an emergent property of complex systems under conditions of informational uncertainty. This multifaceted perspective also allows us to outline current intervention strategies and propose future directions for research, clinical practice, and interdisciplinary modelling.

The present contribution adopts a narrative review approach aimed at synthesizing empirical and theoretical literature across clinical psychology, cognitive science, systems theory, and digital ethics. The literature considered primarily includes peer-reviewed publications published between 2009 and 2025, corresponding to the period in which cyberchondria gained systematic scientific attention. Sources were selected based on their relevance to health anxiety, online health information seeking, metacognitive processes, and digital environmental dynamics. Consistent with the narrative design of the review, no formal systematic inclusion or exclusion protocol was applied.

## 2 Theoretical models: from clinical cognition to complex systems

Initial conceptualizations of cyberchondria emerged within the framework of clinical cognitive-behavioral theory, which situates it as a manifestation of health anxiety driven by dysfunctional beliefs and misinterpretations of bodily sensations (Taylor and Asmundson, 2004; Starcevic and Berle, 2013). According to this model, individuals with heightened health-related vigilance and low tolerance for ambiguity tend to catastrophize benign symptoms and seek excessive reassurance, behaviors that may offer transient relief but ultimately sustain the anxiety cycle (Abramowitz and Braddock, 2011).

Cyberchondria has also been linked to metacognitive dysfunctions, particularly beliefs about the uncontrollability and danger of one’s own thoughts (Fergus and Spada, 2017). This perspective aligns with Wells’ metacognitive model, in which perseverative thinking styles such as worry and rumination are sustained by maladaptive beliefs about cognition itself. In this view, online searching becomes a cognitive coping strategy that paradoxically maintains distress.

More recent approaches have incorporated intolerance of uncertainty (IU) and anxiety sensitivity as core vulnerability factors (Carleton et al., 2007; Schenkel et al., 2021). IU, defined as a dispositional tendency to react negatively to uncertain situations, is particularly relevant in the digital context, where information is not only abundant but often ambiguous, contradictory, or unverifiable (Dugas et al., 1998). These cognitive vulnerabilities interact with the hyper-availability of online content, creating conditions that facilitate recursive loops of uncertainty and reassurance-seeking.

To account for these dynamics, some researchers have applied complexity theory and nonlinear systems models to health-related cognition and affective instability (Olthof et al., 2020; Piccirillo et al., 2024; Pezard and Nandrino, 2001). In this perspective, complexity science offers a crucial framework for capturing the multifaceted dynamics encountered in mental phenomena (Smit and Derksen, 2015).

From this viewpoint, cyberchondria is not reducible to linear cause-and-effect patterns but emerges from interactive feedback loops between psychological traits (e.g., IU, OCD traits), environmental factors (e.g., digital architectures), and contextual triggers (e.g., health-related news, symptoms, or stress). Emergence, a key concept in complexity science, refers to the spontaneous formation of novel and unpredictable patterns from the interaction of simpler components. Such phenomena cannot be predicted or explained by examining the individual components of a system in isolation but instead emerge from the interactions among them (Morin, 2011; Bertalanffy, 2015). A complexity-based perspective is therefore well suited to critically challenge unilateral explanatory models, allowing human functioning to be conceptualized as embedded within a polysystemic network in which neurological, biological, psychological, social, and anthropological dynamics intersect. In this way, complexity theory offers a useful lens for understanding how cyberchondria may arise idiosyncratically across individuals and contexts. Thus, a holistic approach to the study of cyberchondria, as opposed to a unilateral diagnostic perspective, may more effectively integrate diverse domains and risk factors (Yang et al., 2022) while accounting for its multifaceted impact on quality of life, thereby suggesting the adoption of a more comprehensive framework in clinical practice (Yang and Xu, 2025).

In particular, the notion of informational entropy, borrowed from information theory, has been invoked to describe how excessive exposure to conflicting information may lead to disorganization of cognitive appraisal systems. Rather than resolving uncertainty, this surplus of raw data, when insufficiently contextualized, may exacerbate epistemic instability, destabilizing existing schemas and reinforcing recursive anxiety-search cycles.

Information overload, misinformation and a lack of semantic context can indeed undermine our ability to make sense of the world, especially in the contemporary information environment, in which collective well-being largely depends on the effective governance of the information cycle (Floridi, 2009; Floridi, 2011; Floridi, 2014).

In the context of cyberchondria, digital vulnerability manifests as a heightened tendency to amplify anxiety and health-related worries driven by continuous exposure to unfiltered medical information. Many patients turn to the Internet for medical information, often feeling confident that it is accurate and reliable, even though this is not always the case (de Boer et al., 2007).

Cyberchondria may thus be conceptualized as an emerging phenomenon within a complex adaptive system, shaped by the nonlinear interaction between individual vulnerabilities, cognitive processes, and the digital environment. It emerges from a continuous, reciprocal interaction in which mind, technology, and online information co-constitute one another, generating maladaptive patterns of behavior that call for targeted clinical interventions and robust strategies for digital literacy.

Thus, an integrative model of cyberchondria requires bridging clinical cognitive theories with interdisciplinary systems approaches to explain not only what individuals believe and do, but also how self-perpetuating loops of cognitive and behavioral dysregulation emerge through dynamic interaction with complex and unstable informational environments.

Across these perspectives, three interrelated mechanisms consistently emerge: intolerance of uncertainty as a dispositional vulnerability, algorithmic amplification within digital environments, and recursive reassurance-seeking cycles that temporarily reduce distress while reinforcing long-term anxiety. Conceptualizing cyberchondria through the interaction of these mechanisms allows for a more cohesive understanding of its clinical and systemic dynamics.

## 3 Clinical features and behavioral manifestations

Cyberchondria is characterized by a set of behavioral and emotional patterns that go beyond ordinary concerns about health. Individuals affected typically engage in repetitive and prolonged online searches for medical symptoms, often spending hours navigating through websites, forums, and symptom-checker platforms. This behavior is not purely informational; it becomes compulsive and closely linked to escalating emotional distress (McMullan et al., 2019; Vismara et al., 2020).

A hallmark of cyberchondria is the failure of reassurance: instead of alleviating concerns, each search tends to intensify anxiety and reinforce the urge to seek further information. This negative reinforcement loop results in a short-lived sense of relief, which is quickly replaced by new doubts, leading to renewed searching (Starcevic et al., 2020; Vismara et al., 2020). A typical scenario begins with a benign symptom, such as a headache or fatigue, and escalates into the fear of having a severe illness, such as a brain tumor or a neurodegenerative condition.

The emotional correlates include acute anxiety, panic, irritability, and obsessive preoccupation with the possibility of harboring a serious but undiagnosed disease. These reactions are often disproportionate to objective medical findings and persist despite professional reassurance or negative diagnostic results. Rather than feeling reassured, individuals may distrust physicians and seek additional, or alternative, “correct” information online (McMullan et al., 2019; Starcevic et al., 2020).

From a nosographic perspective, cyberchondria overlaps with health anxiety, obsessive-compulsive disorder (OCD), and somatic symptom disorder, but is distinguished by its digital context, which offers instant and unfiltered access to infinite, often contradictory, medical content. This environment enables compulsive patterns to unfold in an unregulated, high-stimulus ecosystem, reinforcing cognitive biases and intolerance of uncertainty (McMullan et al., 2019; Starcevic et al., 2020).

Importantly, cyberchondria exists along a severity spectrum. In more severe cases, it may result in frequent medical consultations, avoidance of care, excessive health-related expenses, or conflictual interpersonal dynamics. In other cases, it remains subclinical yet still undermines well-being and daily functioning. During the COVID-19 pandemic, a marked increase in cyberchondriac behavior was observed, attributed to heightened health anxiety and fear of infection, further validating the role of environmental stressors in exacerbating vulnerability (Boysan et al., 2022).

In sum, cyberchondria can be understood as a behavioral syndrome of digital compulsivity, underpinned by anxiety, cognitive biases, and a progressively dysfunctional relationship with both medical information and professional care.

## 4 Cognitive biases and neuropsychological dimensions

Cyberchondria is not only a behavioral and emotional phenomenon, but is also deeply rooted in cognitive distortions, metacognitive dysfunctions, and neuropsychological vulnerabilities that significantly influence how individuals perceive, interpret, and react to health-related stimuli.

One of the most consistently implicated mechanisms is confirmation bias: the tendency to favor information that supports preexisting fears while dismissing benign or contradictory evidence. During online symptom searches, individuals with cyberchondria frequently ruminate on worst-case explanations, interpreting ambiguous symptoms as signs of serious illness (McMullan et al., 2019; Starcevic et al., 2020). This bias is further amplified by the availability heuristic, whereby emotionally salient and memorable conditions, such as cancer or stroke, come to mind more readily and are perceived as more probable.

Another key factor is selective attention to threatening stimuli. Individuals with high health anxiety demonstrate attentional bias toward bodily sensations and health-related information, especially when immersed in digitally rich environments (Starcevic et al., 2020; Norr et al., 2015). This hypervigilance elevates the perceived severity of minor symptoms and fuels repeated checking and reassurance-seeking behavior.

The interpretation of bodily sensations in cyberchondria tends toward catastrophizing. Minor or transient sensations, such as fatigue, dizziness, or muscle twitches, are often construed as harbingers of life-threatening illness. This pattern of catastrophic interpretation is linked to cognitive inflexibility, a diminished ability to adjust cognitive appraisals, and a poor tolerance of uncertainty (Schenkel et al., 2021; de Frota Lisboa Pereira Souza et al., 2024).

Neuropsychological profiles associated with cyberchondria suggest impairments in executive functions, particularly those involved in inhibitory control, emotional regulation, and decision-making. Though direct empirical studies are sparse, related conditions such as OCD and somatic symptom disorder point to dysfunctional frontal-limbic circuitry, which may impair individuals’ capacity to disengage from intrusive thoughts or inhibit compulsive checking behaviors (Boysan et al., 2022). Foundational work on executive functions highlights their critical role in mental flexibility, working memory, and inhibitory control, faculties likely compromised in cyberchondriac individuals (Diamond, 2013; Suchy, 2009).

Moreover, metacognitive dysfunctions are central to sustaining cyberchondria. Individuals tend to overestimate the value of repeated searching (“If I check again, I will finally be sure”) and underestimate its emotional cost, reinforcing a maladaptive cognitive loop (Vismara et al., 2020). Recent structural models indicate that cognitive bias, health-related metacognition, and emotion dysregulation fully mediate the relationship between personality traits and cyberchondria (Nasiri et al., 2023).

In sum, cognitive and neuropsychological mechanisms, including attentional bias, confirmation bias, catastrophizing, impaired executive control, and dysfunctional metacognition, converge to create a cognitive environment in which cyberchondria thrives. These processes are dynamic and interactive, shaping how individuals process uncertainty in a hyperconnected and informationally unchecked environment.

## 5 The digital ecosystem: from search engines to AI-driven amplification

The behavioral and cognitive dynamics of cyberchondria cannot be fully understood without examining the digital ecosystem in which they unfold. Unlike traditional health anxiety, cyberchondria is inseparable from the architecture of the internet, including search engines, health websites, forums, and increasingly, AI-driven tools. These platforms do more than facilitate information access; they modulate users’ perception of risk, amplify uncertainty, and reinforce compulsive searching.

At the core lies the algorithmic structure of search engines. When users enter vague symptoms (e.g., “headache and fatigue”), algorithms often surface high-engagement or sensational content, frequently emphasizing worst-case scenarios. This search-result bias increases threat salience and intensifies emotional responses, transforming confirmation bias into a digitally reinforced loop (McMullan et al., 2019; Starcevic et al., 2020). The phenomenon mirrors the theory of the “filter bubble” (Pariser, 2011) and echo chambers, where personalization over time limits exposure to disconfirming information, further entrenching digital confirmation bias (Purba et al., 2024).

Moreover, health forums and social media often function as digital echo chambers, where anecdotal symptom narratives are amplified and anxiously shared. These peer-driven interactions simulate reassurance-seeking behaviors but lack clinical validation, ultimately reinforcing maladaptive health beliefs and escalating anxiety (Vismara et al., 2020; Bizzotto et al., 2023).

A growing concern is the rise of AI-powered symptom checkers and chatbots. Although designed to support self-diagnosis, these tools may inadvertently increase anxiety by suggesting rare or severe diagnoses, especially when lacking transparent contextualization. User trust in these systems is often influenced by their perceived objectivity, despite limitations in accuracy and transparency (Mahlknecht et al., 2023; Woodcock et al., 2021).

Recent developments in generative AI have introduced systems that allow users to integrate personal medical records and health data to generate increasingly personalized health-related responses, raising important ethical concerns. From a strictly bioethical standpoint, the incorporation of sensitive health data into proprietary platforms inherently poses fundamental questions about data sovereignty and privacy.

The speed and volume of digital information contribute to informational overload, elevating subjective uncertainty. The Entropy Model of Uncertainty explains that when information surpasses people’s integrative capacity, internal entropy increases, leading to anxiety, confusion, and cognitive fragmentation (Hirsh et al., 2012).

## 6 Clinical consequences and the medical relationship

The clinical consequences of cyberchondria extend well beyond the individual’s internal experience of anxiety. One of the most significant impacts concerns the doctor-patient relationship, which may be strained or disrupted by distrust, over-consultation, or conflict over diagnostic interpretations.

Since its very origins, bioethics has been committed to rethinking the patient-physician relationship, with particular attention to the transformations brought about by modern technology and its derivatives. In this context, the disruptive impact of AI, which is now forcefully entering clinical practice, further complicates the already challenging task of achieving adequate communication, which must always remain at the heart of the healthcare profession (Sandeep et al., 2024; Merlo et al., 2026), as also highlighted by the recent development of care ethics (Adorno, 2019). Neglecting the importance of communication in the patient-physician relationship means undermining the ethical foundation of caregiving. Hence, a critical appraisal of the new possibilities offered by AI in healthcare is necessary (Giacobello, 2025).

Individuals experiencing cyberchondria often approach medical consultations after extensive online searching, bringing a predefined set of diagnostic hypotheses shaped by algorithmic results and nonclinical sources (Starcevic et al., 2020). When their expectations are not validated by the physician, frustration and dissatisfaction may arise, potentially undermining the therapeutic alliance.

Paradoxically, the search for reassurance, which drives much of the online behavior in cyberchondria, can lead to increased health service utilization, including unnecessary consultations, laboratory tests, specialist referrals, and imaging procedures (McMullan et al., 2019). This contributes to overmedicalization, increases healthcare costs, and poses a risk of iatrogenic harm, including false positives and overtreatment. Moreover, recent cross-sectional data from Poland indicate that higher cyberchondria severity is significantly predictive of increased use of family physician visits, emergency services, hospital admissions, and even alternative medicine, after controlling for sociodemographic and health variables (Kobryn and Duplaga, 2024). At the same time, some individuals, after repeated negative evaluations, may avoid seeking professional care altogether, believing that no doctor understands or will correctly diagnose their condition (Schenkel et al., 2021). This oscillation between hyper-consultation and medical avoidance generates a profound clinical dilemma. In patients with chronic or medically unexplained symptoms, cyberchondria may become a stable maintaining factor that interferes with treatment compliance and continuity of care.

In clinical practice, physicians frequently encounter patients whose interpretations of symptoms are rooted in online information. This can create a power imbalance, in which the clinician’s expertise is challenged by what the patient perceives as a more accurate or objective representation of reality found online. At worst, this dynamic encourages defensive medicine, as clinicians feel compelled to accommodate patients’ fears to preserve rapport, even when medically unwarranted. In other instances, it leads to breakdowns in communication, especially if physicians dismiss patients’ online-derived concerns too abruptly (Vismara et al., 2020). A qualitative study among general practitioners confirms their experience of professional frustration and uncertainty when managing cyberchondriac patients (Wangler and Jansky, 2020).

The emotional toll on clinicians should not be underestimated. Repeated encounters with cyberchondriac patients may provoke feelings of frustration, helplessness, or even professional inadequacy. This dynamic calls for a more nuanced approach to patient communication, grounded in validation, psychoeducation, and collaborative exploration of reliable online sources during consultations. In this light, cyberchondria emerges not just as a patient problem, but as a relational and systemic challenge in contemporary clinical care.

Recent work has also emphasized the need for structured clinical guidelines to help health professionals identify and manage cyberchondria. Suggested tools include screening instruments, differential diagnostic strategies (to distinguish cyberchondria from OCD or somatic symptom disorder), and communication protocols designed to reduce confrontation and foster trust. Furthermore, incorporating digital health literacy into clinician training can empower professionals to better navigate patients’ online narratives without undermining their autonomy (Lu et al., 2024).

In sum, cyberchondria has multi-level clinical implications: it affects patient well-being, distorts symptom perception, inflates healthcare utilization, and complicates the doctor-patient relationship, thereby demanding a shift toward relationally attuned and digitally informed clinical practice.

## 7 Evidence-based interventions and clinical recommendations

The growing recognition of cyberchondria as a distinct clinical phenomenon has led to the development of targeted psychological interventions, primarily adapted from cognitive-behavioral therapy (CBT) and health anxiety treatment protocols. Although cyberchondria is not yet a formal diagnosis in DSM-5 or ICD-11, therapeutic models increasingly address its unique features, particularly compulsive online searching, reassurance-seeking, and intolerance of uncertainty (Mentzoni et al., 2011; Vismara et al., 2021).

Cognitive-behavioral therapy remains the most studied and effective approach. Treatment typically focuses on identifying and restructuring maladaptive beliefs about health and illness, reducing reassurance-seeking behaviors, and increasing tolerance for uncertainty (Mentzoni et al., 2011; Mathes et al., 2018). In the context of cyberchondria, CBT protocols often include modules on digital behavior awareness, helping individuals monitor and gradually reduce their health-related internet use. Behavioral experiments, cognitive restructuring, and exposure techniques are used to test catastrophic interpretations of bodily symptoms and challenge the perceived necessity of repeated searches (Mathes et al., 2018; Cooper et al., 2017).

Emerging research supports the efficacy of metacognitive therapy (MCT) as well. This approach targets dysfunctional metacognitive beliefs such as “If I keep searching, I’ll eventually feel reassured” or “Not knowing is dangerous,” which are central to the persistence of cyberchondria (Nordahl et al., 2025). Interventions aim to modify beliefs about thought processes, rather than the content of thoughts, offering a higher-level cognitive intervention for compulsive checking behaviors. A randomized controlled trial by Nasiri et al. (2023) found that metacognitive beliefs significantly predicted treatment response in individuals with cyberchondria and high health anxiety.

Psychoeducation is a critical component in both CBT and general clinical practice. Patients benefit from learning about the dynamics of online information, including algorithmic bias, anecdotal unreliability, and cognitive distortions. This knowledge empowers patients to make more informed and reflective decisions about when and how to seek health information online (Vismara et al., 2021).

A promising avenue is the development of digital tools, such as apps or browser extensions, that help track and limit symptom-checking behaviors. These interventions provide real-time feedback, support self-monitoring, and facilitate gradual exposure to uncertainty. While still in early stages of development and validation, such tools may complement traditional therapy by integrating behavior change strategies into daily online habits (Muse et al., 2010).

From a systems-level perspective, healthcare providers and public institutions can mitigate cyberchondria through digital health literacy campaigns. These include teaching patients to evaluate the credibility of sources, distinguish between reliable and misleading content, and use institutional or peer-reviewed platforms instead of commercial or sensationalist websites (Starcevic et al., 2020).

Clinicians may also benefit from training in communication strategies specifically tailored to cyberchondria. These include validating patient concerns, co-browsing credible sources during consultations, and collaboratively building trust around medical knowledge. Moreover, tools like the Cyberchondria Severity Scale (CSS) can assist with early detection and monitoring of problematic internet use related to health concerns (McElroy and Shevlin, 2014).

In summary, effective interventions for cyberchondria must operate at multiple levels: intrapsychic (beliefs and thoughts), behavioral (search habits), relational (doctor-patient interaction), and systemic (digital ecosystems). A combination of CBT, metacognitive strategies, psychoeducation, and digital literacy efforts appears most promising for effective prevention and treatment.

## 8 Ethical and societal dimensions

The rise of cyberchondria in the digital age poses not only clinical and psychological challenges but also significant ethical and societal implications. At the core lies a fundamental question: Who is responsible for the mental health consequences of unrestricted access to health information online?

The autonomy of the individual, a foundational principle in modern medicine, is complicated by the opacity of algorithmic systems that curate and prioritize health-related content (Floridi, 2016a). Users may believe they are exercising free and informed choice, while they are being nudged toward emotionally salient, popular, or fear-inducing information based on opaque and commercially driven digital architectures (Floridi, 2016b; Sandeep et al., 2024). This raises profound questions about the ethics of information design, especially when emotionally vulnerable individuals are exposed to uncontextualized or alarmist content that may increase distress or reinforce compulsive health-checking behaviors (Saracini et al., 2025).

Moreover, a tension exists between the right to access medical knowledge and the cognitive capacity to interpret it appropriately. While the democratization of health information is a key achievement of the digital age, it also carries the risk of epistemic overload, a state in which individuals are unable to distinguish credible evidence from misinformation or commercially biased sources (O’Neill, 2002; Meppelink et al., 2019). This condition resonates with broader critiques of the “transparency society,” in which excessive visibility and continuous exposure to information may undermine interpretative depth and reflective judgment (Han, 2015). In the context of cyberchondria, this confusion can foster false beliefs, excessive self-monitoring, and dangerous self-diagnosis, often without professional guidance.

The responsibilities of digital platforms, including search engines, AI-powered chatbots, and symptom checkers, are increasingly under ethical and legal scrutiny. Should these systems be required to implement safeguards against the amplification of catastrophic interpretations? Should they highlight uncertainty, flag unverified or anecdotal content, or redirect users exhibiting health-anxious patterns to reliable sources? These questions remain largely unregulated, despite their potential impact on public mental health and trust in healthcare systems (Sandeep et al., 2024; Goktas and Grzybowski, 2025). Technology companies should be held to more stringent standards of algorithmic ethics, including the adoption of safeguards to mitigate psychopathological risks and the implementation of responsible recommendation protocols. This underscores the urgent need for an integrated framework of algorithmic ethics, digital policy regulation, and user awareness promotion to protect vulnerable individuals within the digital health ecosystem (Li et al., 2025; Floridi and Taddeo, 2025).

From this perspective, digital ethics can contribute to mitigating cyberchondria at multiple levels. First, the ethical platform design is essential to reduce exposure to alarmist content through transparent algorithms and responsible recommendation strategies. A further crucial dimension concerns the quality of health information, which must be ensured by prioritizing reliable and evidence-based sources. Content producers also bear responsibility for avoiding sensationalistic communication, acknowledging informational limits and maintaining up-to-date accuracy. Moreover, digital education is crucial to strengthening critical thinking and fostering awareness of cognitive and informational biases. An ethical approach oriented toward care and individual wellbeing should ultimately support the integration of human assistance with digital tools, to help contain anxiety and prevent pathological escalation. Ultimately, responsible governance is necessary to establish clear standards and mechanisms of accountability for platforms and information systems (Floridi et al., 2018; Floridi and Cowls, 2019; Floridi, 2023). These governance principles are consistent with recent European ethical frameworks for digital health, which emphasize transparency, user protection, proportionality, and equitable access as core safeguards in digitally mediated healthcare environments (Council of Europe, 2023).

Importantly, digital vulnerability is unequally distributed. Individuals with low health literacy, high anxiety, limited access to care, or preexisting psychopathological conditions are disproportionately affected. These users are less equipped to appraise digital content critically and more susceptible to algorithmically reinforced health anxiety (Meppelink et al., 2019; Pérez-Escolar and Canet, 2023). Thus, cyberchondria may widen health disparities, not only in terms of mental distress but also by eroding confidence in medical authority and increasing reliance on non-evidence-based sources.

In this context, cyberchondria assumes particular significance within contemporary bioethical discourse, which, especially in its recent developments, has placed the concept of vulnerability at the center, framing it as a global and multidimensional phenomenon. Anthropological vulnerability refers to the general human condition as fragile and precarious due to its radical finitude and its fundamental dependence on others; special vulnerability, by contrast, denotes a condition arising only in specific circumstances and precisely for this reason, demands collective efforts to promote measures aimed at counteracting it (ten Have, 2015; ten Have, 2016; Ten Have and Gordijn, 2021). Cyberchondria can be situated within this second category, as a form of psychological and digital vulnerability that emerges under specific conditions of extensive online health information seeking and algorithmically mediated content, which may amplify emotional distress and uncertainty.

From a bioethical standpoint, the global well-being of individuals in today’s digital ecosystem (Burr et al., 2020; Ding et al., 2025) requires collaboration between clinicians, educators, policymakers, and developers to define a shared framework of digital responsibility. This should include:

- Integrating digital health literacy into public education and clinical practice.
- Developing ethical guidelines for AI in health information delivery.
- Equipping clinicians to engage with patients’ online health behaviors without judgment.
- Promoting transparency and accountability in the architecture of health platforms and algorithms.

In conclusion, the ethical and societal dimensions of cyberchondria demand an interdisciplinary response that bridges health psychology, digital ethics, public policy, and technological governance. If left unaddressed, cyberchondria may not only increase individual suffering but also destabilize the fragile equilibrium between digital autonomy, clinical authority, and public trust.

## 9 Discussion

The phenomenon of cyberchondria presents a complex and multidimensional challenge that a single explanatory model cannot fully capture. The evidence synthesized across clinical, cognitive, digital, and ethical domains suggests that cyberchondria should be understood not merely as an anxiety disorder in the digital era, but as an emergent property of a system in which psychopathological vulnerability, technological architecture, and informational instability interact in nonlinear and amplifying ways.

Clinically, the literature confirms that cyberchondria is strongly associated with health anxiety, intolerance of uncertainty, obsessive-compulsive tendencies, and metacognitive dysfunctions (McMullan et al., 2019; Nordahl et al., 2025). Behaviorally, it manifests as compulsive online symptom-checking, with search behavior serving both as a maladaptive coping mechanism and a source of reinforcement for catastrophic health interpretations (Starcevic et al., 2020). From a digital perspective, algorithms, search engine bias, social platforms, and AI-powered symptom checkers exacerbate cognitive vulnerabilities, often without the user’s awareness (Floridi, 2016b; Sandeep et al., 2024). These findings call for a reframing of cyberchondria as not only a psychopathological entity, but also a dynamical and informational phenomenon, in line with recent perspectives from complexity science and information theory. According to the Entropy Model of Uncertainty, anxiety increases as internal cognitive entropy rises, particularly when individuals are confronted with ambiguous or excessive information that they cannot assimilate coherently (Hirsh et al., 2012; Cristín et al., 2022). Cyberchondria exemplifies this process: the more individuals search, the more they encounter conflicting interpretations, rare disease scenarios, and emotionally salient content, amplifying uncertainty rather than resolving it.

Such a system can be described in terms of feedback loops and positive reinforcement dynamics, which are characteristic of complex adaptive systems. The individual mind is not a passive recipient of information but an active processor that responds to instability by engaging in behaviors (e.g., searching) that temporarily reduce anxiety but ultimately destabilize the system further. In this sense, cyberchondria may represent a form of cognitive decoherence, understood as a metaphorical breakdown of informational coherence across competing mental representations. It describes the collapse of stable belief states under informational overload and should not be confused with quantum decoherence in the physical sciences.

From a systems theory perspective, one could model cyberchondria as a system operating near a bifurcation point, where small perturbations (e.g., a minor symptom) can lead to large, nonlinear effects (e.g., panic, compulsive consultation, diagnostic escalation). This interpretation aligns with existing models of mental disorders as instabilities in self-organizing systems, modulated by internal and external informational flows (Friston, 2010). In the present work, complexity theory and informational entropy are proposed primarily as interpretive and heuristic frameworks for conceptualizing cyberchondria as a dynamic, nonlinear system. At the same time, these perspectives may also provide a foundation for future empirical and computational modelling, including simulations of feedback loops and network-based representations of belief updating under uncertainty.

This integrative framework suggests new interdisciplinary paths for research, including:

- Computational modelling of symptom-checking dynamics.
- Simulation of informational entropy thresholds for anxiety escalation.
- Network-based representations of belief updating under uncertainty.
- Experimental studies on AI-user interaction in symptom-checking contexts.

Ultimately, the discussion of cyberchondria must evolve beyond traditional psychopathology toward a systems-level view that encompasses the cognitive, emotional, technological, and epistemological dimensions of health anxiety in the digital age.

### 9.1 Strengths and limitations

This narrative review provides a comprehensive and interdisciplinary exploration of cyberchondria, integrating clinical psychology, cognitive models, systems theory, and digital ethics. By examining the phenomenon through the lenses of complexity theory and informational entropy, the review offers a novel theoretical synthesis that extends beyond traditional psychopathological frameworks. It highlights the recursive nature of digital symptom-checking, the central role of intolerance of uncertainty, and the systemic impact of algorithmic structures on mental health, thereby contributing a broader epistemological, clinical, and societal perspective to the field.

Nonetheless, as a narrative review, this work does not rely on systematic methods for study selection or quality assessment, which may introduce selection bias and limit reproducibility. Moreover, although the theoretical proposals are grounded in the current literature, they remain largely conceptual and require further empirical validation. The diversity of sources and disciplinary perspectives may also result in heterogeneity in the definition and operationalization of key constructs. Future research should address these limitations by adopting systematic review methodologies, developing quantitative and computational models, and conducting experimental and longitudinal studies aimed at testing and refining the proposed theoretical frameworks.

## 10 Conclusion

Cyberchondria represents a paradigmatic expression of the interplay between mental health, digital infrastructures, and informational complexity in the 21st century. While it shares features with health anxiety, obsessive–compulsive behaviors, and somatic symptom disorders, cyberchondria is distinguished by its embeddedness in the digital environment and its recursive feedback loops, which reshape the structure and function of psychological processes. It appears, in fact, that the Internet plays a fundamental role in the lives of individuals who experience anxiety about their health (Baumgartner and Hartmann, 2011).

Within this framework, cyberchondria serves as an illuminating example of how digital technology can play a decisive, albeit not exclusive, role in the emergence and maintenance of psychopathological phenomena. At the same time, cyberchondria also reveals new modes of manifestation of hypochondria, a condition recognized for centuries, highlighting the need to better understand the connections and etiological pathways linking hypochondria, health anxiety, and online health-related searching (Jungmann et al., 2024; Starcevic, 2017). Ultimately, it is now crucial to understand how contemporary digital technologies contribute to health-related concerns and shape individuals’ perceptions of their own health (Yorulmaz et al., 2025), especially considering established evidence linking Internet addiction, online health information-seeking behaviors, and cyberchondria (Deniz, 2025).

The review of empirical studies confirms that cyberchondria is closely associated with intolerance of uncertainty, metacognitive dysfunction, and reassurance-seeking behaviors. These psychological mechanisms are further exacerbated by the algorithmic architecture of online information, which privileges emotional salience over epistemic reliability, and popularity over clinical accuracy (McMullan et al., 2019; Starcevic et al., 2020). As a result, individuals are often caught in a self-reinforcing cycle of symptom-checking, escalating anxiety, and cognitive fragmentation.

Evidence-based interventions, such as cognitive-behavioral therapy, metacognitive strategies, and digital psychoeducation, demonstrate efficacy in reducing the emotional and behavioral impact of cyberchondria. However, their long-term effectiveness depends on addressing not only individual-level dynamics, but also relational, informational, and systemic factors, including the doctor-patient alliance, public health communication, and the ethical governance of digital platforms (Nordahl et al., 2025; Floridi, 2016b).

From a theoretical standpoint, cyberchondria challenges linear and reductionist frameworks of psychopathology. The integration of complexity theory, informational entropy models, and systems neuroscience provides a promising conceptual foundation to reconceptualize anxiety-related disorders as emergent instabilities within saturated and uncertain informational environments (Hirsh et al., 2012; Friston, 2010).

As digital technologies continue to evolve, and as AI-driven tools increasingly participate in medical decision-making, the urgency for interdisciplinary cooperation grows. Mental health professionals, clinicians, educators, policymakers, and technologists must collaborate to co-create an informational ecosystem that supports psychological integrity rather than undermines it.

Cyberchondria may therefore be understood as a complex systems phenomenon, in which unlimited access to online information increases informational entropy, generating anxiety and cognitive stress. This leads to compulsive behaviors of searching for and monitoring symptoms, which fuel positive-feedback loops and mental instability. In this way, mind, technology, and information interact nonlinearly, giving rise to an emerging digital health disorder.

In this sense, cyberchondria is not merely a clinical entity; it is also a mirror of our epistemic condition, in which unrestricted access to information paradoxically breeds uncertainty, disorientation, and distress. Addressing it requires not only therapeutic innovation, but also a critical redesign of the architecture of digital life, rooted in a realistic understanding of human cognitive and emotional limits and informed by principles of psychological safety and epistemic responsibility.

From this perspective, cyberchondria can be considered not only a disorder of the digital age, but also a clinically significant determinant of medical behavior and health outcomes, fully consistent with contemporary models of mind–body interaction in chronic and complex medical conditions.
